# Fatigue following COVID-19 infection is not associated with autonomic dysfunction

**DOI:** 10.1371/journal.pone.0247280

**Published:** 2021-02-25

**Authors:** Liam Townsend, David Moloney, Ciaran Finucane, Kevin McCarthy, Colm Bergin, Ciaran Bannan, Rose-Anne Kenny

**Affiliations:** 1 Department of Infectious Diseases, St James’s Hospital, Dublin, Ireland; 2 Department of Clinical Medicine, School of Medicine, Trinity Translational Medicine Institute, Trinity College Dublin, Dublin, Ireland; 3 The Irish Longitudinal Study on Ageing (TILDA), Trinity College Dublin, Dublin, Ireland; 4 Falls and Syncope Unit, Mercer’s Institute for Successful Ageing, St James’s Hospital, Dublin, Ireland; 5 Department of Medical Physics and Bioengineering, Mercer’s Institute for Successful Ageing, St James’s Hospital, Dublin, Ireland; Universidad Miguel Hernandez de Elche, SPAIN

## Abstract

**Background:**

The long-term clinical and physiological consequences of COVID-19 infection remain unclear. While fatigue has emerged as a common symptom following infection, little is known about its links with autonomic dysfunction. SARS-CoV-2 is known to infect endothelial cells in acute infection, resulting in autonomic dysfunction. Here we set out to test the hypothesis that this results in persistent autonomic dysfunction and is associated with post-COVID fatigue in convalescent patients.

**Methods:**

We recruited 20 fatigued and 20 non-fatigued post-COVID patients (median age 44.5 years, 36/40 (90%) female, median time to follow up 166.5 days). Fatigue was assessed using the Chalder Fatigue Scale. These underwent the Ewing’s autonomic function test battery, including deep breathing, active standing, Valsalva manoeuvre and cold-pressor testing, with continuous electrocardiogram and blood pressure monitoring, as well as near-infrared spectroscopy-based cerebral oxygenation. 24-hour ambulatory blood pressure monitoring was also conducted, and patients completed the generalised anxiety disorder-7 questionnaire. We assessed between-group differences in autonomic function test results and used unadjusted and adjusted linear regression to investigate the relationship between fatigue, anxiety, and autonomic test results.

**Results:**

We found no pathological differences between fatigued and non-fatigued patients on autonomic testing or on 24-hour blood pressure monitoring. Symptoms of orthostatic intolerance were reported by 70% of the fatigued cohort at the time of active standing, with no associated physiological abnormality detected. Fatigue was strongly associated with increased anxiety (p <0.001), with no patients having a pre-existing diagnosis of anxiety.

**Conclusions:**

These results demonstrate the significant burden of fatigue, symptoms of autonomic dysfunction and anxiety in the aftermath of COVID-19 infection, but reassuringly do not demonstrate pathological findings on autonomic testing.

## Introduction

The COVID-19 pandemic, caused by SARS-CoV-2 infection, has dominated world news since it first emerged in the city of Wuhan, China, in December 2019 [1]. The clinical features of acute infection have been well-described, ranging from mild disturbance in taste and smell to progressive shortness of breath and respiratory failure [2,3]. Similarly, the pathological changes during acute disease are also well-described, with development of coagulopathy and myeloid cell dysregulation in severe disease [4,5]. The sequalae and complications of COVID-19 are beginning to be described clinically; however, the underlying pathophysiology is poorly understood. There are a multitude of symptoms that persist after resolution of acute illness, giving rise to so-called *long COVID* [6]. The primary complaint appears to be persistent fatigue and we have previously demonstrated a high prevalence of fatigue in convalescent COVID-19 patients [7,8].

Autonomic dysfunction most commonly manifests within the cardiovascular system with tachycardia, hypotension and vasovagal syncope [9]. It has been described following a myriad of infections, including viral, bacterial and parasitic [10,11]. However, the mechanisms behind this dysfunction remain unclear. There is limited data describing autonomic dysfunction following coronavirus infection. A recent case report has demonstrated the presence of postural orthostatic tachycardia syndrome (POTS) following SARS-CoV-2 infection, as defined by an increase in heart rate >30 beats per minute following head-up tilt test [12]. Furthermore, pathological changes seen in acute infection are known to affect autonomic function. Endothelial cells can be directly infected by the SARS-CoV-2 virus as they express the ACE2 receptor, which is the binding site for the infecting virus [13]. Endothelial dysfunction in acute COVID-19 has been associated with multi-organ dysfunction [14,15]. Endothelial cell infection has also been described in previous pandemic coronaviruses such as MERS and SARS-CoV-1 [16]. After the SARS-CoV-1 outbreak, a small subgroup of patients described palpitations and tachycardia. However, no obvious cardiac abnormalities were detected [17]. Persistent tachycardia was noted in over 1/3 of patients 30 days after their initial infection, but again no pathological process has been identified [18].

We set out to investigate the presence of autonomic dysfunction following SARS-CoV-2 infection and its relationship with post-COVID fatigue.

## Methods

### Study setting and participants

This study was carried out in the falls and syncope unit at St James’s Hospital, Dublin, Ireland. Patients were recruited via the post-COVID-19 clinic at the same hospital between August and October 2020. The inclusion criteria were positive SARS-CoV-2 RT-PCR test at time of acute illness and patients aged >18 years of age. Patients were excluded if they were in receipt of any medication that directly affected heart rate or blood pressure, such as beta blockers and anti-hypertensives, or were unable to complete any part of the assessment. Demographic data was recorded, and severity of initial infection was graded based on requirement for hospitalisation. Proportions of healthcare workers were matched between fatigued and non-fatigued cohorts. The population selected was representative of the population seen in our outpatient clinic and those reported from elsewhere [19]. A clinical frailty score was assessed for each participant, using the Fried frailty score [20]. Informed consent was obtained from all participants in the current study in accordance with the Declaration of Helsinki [21]. Ethical approval for the current study was obtained from the Tallaght University Hospital/St James’s Hospital Joint Research Ethics Committee (reference REC 2020–04 (01)) and informed written consent was obtained from all participants.

### Fatigue assessment

Fatigue was assessed using the Chalder Fatigue Scale (CFQ-11) at time of outpatient appointment and at time of autonomic testing [22,23]. Participants answer eleven questions in relation to physical and psychological fatigue, with particular reference to the past month in comparison to their pre-COVID-19 baseline. Responses were measured on a Likert scale (*Better than usual* = 0, *No worse than usual* = 1, *worse than usual* = 2, *much worse than usual* = 3). From this a global score was constructed out of a total of 33, with a score of 11 indicating no difference from prior to infection, reflecting answers of *no worse than usual* [24]. We also used a dichotomous fatigue case definition, whereby scores 0 and 1 (*Better than usual/No worse than usual*) are scored a zero and scores 2 and 3 (*Worse than usual/Much worse than usual*) are scored a 1. A total of four or higher meets the case definition of fatigue [24–27].

The generalised anxiety disorder-7 (GAD7) questionnaire was also completed by all patients. This is a well-validated screening tool and symptom severity measure for the four most common anxiety disorders [28–30].

### Autonomic testing

Ewing’s autonomic function test battery was performed, comprising of deep breathing, active stand, Valsalva manoeuvre, and cold pressor testing [31]. A 12-lead electrocardiogram (ECG) (ELI 380, Mortara, Welch Allyn) was obtained prior to testing, with a 5-lead ECG providing continuous ECG monitoring during testing. This was used to derive measures of heart rate variability (HRV) during supine resting and provocative testing. A detailed description of continuous blood pressure (BP) monitoring using beat-to-beat systolic BP, diastolic BP and pulse rate using the volume clamp method (Finometer NOVA, FMS Medical Systems, Arnheim, Netherlands) during autonomic testing is available elsewhere [32]. Briefly, a pressure cuff was applied to the patient (left hand, middle finger) with height correction. Physiocal^™^ and brachial calibration was applied during patient setup.

Concurrent continuous cerebral oxygenation measures using near-infrared spectroscopy (NIRs) was also recorded (Portalite, Artinis Medical Systems B.V., Elst, Netherlands) throughout to derive a tissue oxygenation/saturation index (TSI). A NIRs sensor was placed on the left side of the forehead to measure frontal lobe cerebral oxygenation, centred at 3cm lateral and 3.5cm above the nasion with an opaque head bandage applied to provide environmental light protection.

While lying supine, the patient was instructed to take controlled deep breaths in and out at a rate of 6 breaths per minute. Following two minutes of sitting, patients then rested in the supine position for ten minutes. This period was used to derive measures of HRV. Patients were then asked to stand as quickly as possible under the supervision of a researcher and continued to stand for five minutes. During this period, they were asked to report any symptoms of light-headedness, dizziness, palpitations or chest discomfort. Subsequently, the patient was seated in a chair for two minutes. They were then instructed to perform a Valsalva manoeuvre by forced expiration against a disposable air flow restrictor and directed to maintain an expiratory pressure of 40mmHg for a minimum of 8 seconds, recording phase I (reduced HR, increased BP at initiation) to phase IV (normal physiological overshoot of blood pressure after completion of Valsalva manoeuvre). This procedure was repeated twice. Finally, the patient was asked to place their hand in a basin of iced water until the diastolic BP rose from baseline by 10mmHg or for one minute, whichever occurred earliest.

### Analysis of variables

The following data were extracted by a bioengineer blinded to the patient diagnosis: heart rate responses to deep breathing (expiratory/inspiratory ratio), active stand heart rate ratio (30^th^ beat/15^th^ beat ratio), R-R interval, and the Valsalva ratio (R-R post / R-R during). The blood pressure response to active stand, Valsalva and cold pressor testing was also recorded and analysed using proprietary software (Novascope V 1.10) and custom-written software in MATLAB R2017a (The Math Works, Inc., MATLAB, Version 2017a, Natick, MA) as described elsewhere [33]. Furthermore, the change of blood pressure and heart rate from baseline to 10 seconds and 20 seconds after active standing was recorded, as was the change from 10 seconds to 20 seconds. This latter measurement has been associated with increased mortality and frailty [34]. Blood pressure at 40 seconds after active standing was also recorded to account for impaired BP and HR stabilisation, which is associated with falls, frailty, impaired cognitive performance and mortality [35–37]. There were four TSI measurements recorded during active standing: baseline prior to active stand, initial nadir (within first 30 seconds of standing), maximum overshoot, and nadir at any timepoint during stand.

A 24-hour ambulatory blood pressure monitor (24H ABPM) was recorded for each patient. This allowed extraction of mean and standard deviation of systolic BP, diastolic BP, mean arterial pressure (MAP), pulse pressure (PP) and heart rate (HR) over 24 hours, as well as mean values for day and night-time.

### Statistical analysis

All statistical analysis was carried out using STATA v15.0 (Stata Statistical Software, College Station, TX StataCorp LP.) and statistical significance considered p <0.05. Descriptive statistics are reported as means with standard deviations (SD) and medians with interquartile ranges (IQR) as appropriate. Univariate analysis was performed on important demographic variables and autonomic test results to examine differences between fatigued and non-fatigued individuals, using t-test (*t*), Wilcoxon rank-sum (*z*) and Chi-squared test (*χ*^*2*^) as appropriate following Shapiro-Wilk testing for normality. Linear regression, with CFQ-11 score as the dependent variable, was used to investigate the relationship with autonomic function testing results under both unadjusted and adjusted (age, sex, clinical frailty score, and need for admission during acute infection) conditions. Results are reported as β coefficients and confidence intervals (CIs) with associated p-values. Bonferroni correction was applied to correct for multiple testing.

## Results

Forty patients were recruited, twenty of whom met the case definition for fatigue and twenty who did not. The median age was 44.5 years (IQR 33–51), while the median time to follow up was 166.5 days (IQR 154.5–179). There were no differences in demographics, body mass index, medical history or clinical frailty status between the cohorts (Table 1). No patients had a pre-existing diagnosis of depression or anxiety, nor were any patients in receipt of anxiolytic or anti-depressant therapy. None of the patients had required admission to the intensive care unit during acute infection. All patients had been in full-time employment prior to SARS-CoV-2 infection; 20 (20/20, 100%) non-fatigued patients had returned to work, while 13 of the fatigued cohort (13/20, 65%) had returned to full-time employment following infection.

**Table 1:** Cohort demographics and baseline measurements.

	Total Cohort (n = 40)	Non-Fatigued (n = 20)	Fatigued (n = 20)	Statistic
Age, years, median (IQR)	44.5 (35–51)	45 (39.5–53)	44 (32.5–48)	*t* = 1.5, *p* = 0.94
Sex, female, n (%)	36 (90)	18 (90)	18 (90)	Χ^*2*^ = 0.00, *p* > 0.99
Admitted, n (%)	7 (17.5)	2 (10)	5 (25)	Χ^*2*^ = 1.56, *p* = 0.21
HCW, n (%)	38 (95)	20 (100)	18 (90)	Χ^*2*^ = 2.11, *p* = 0.15
Duration from COVID diagnosis to assessment, days, median (IQR)	166.5 (154.5–179)	174.5 (162–183.5)	156.5 (134–173)	*t* = 3.12, *p* = 0.99
Clinical Frailty Score, median (IQR)	1 (1–2)	1 (1–2)	1 (1–2)	*z* = -0.32, *p* = 0.75
Co-morbidities, n, median (IQR)	0 (0–1)	0 (0–0.5)	1 (0–1)	*z* = -1.84, *p* = 0.07
Co-medications, n, median (IQR)	0.5 (0–1)	0 (0–1)	1 (0–1)	*z* = -1.4, *p* = 0.16
BMI, kg/m^2^, median (IQR)	24.1 (22.1–28.1)	23.7 (22.2–25.4)	25.9 (22.0–28.3)	*z* = -0.66, *p* = 0.51
Fatigue score, median (IQR)	15.5 (11–23)	11 (11–13)	23 (20–26.5)	*z* = -5.47, *p* < 0.001
Return to work, n (%)	33 (82.5)	20 (100)	13 (65)	Χ^2^ 4.33, *p* = 0.04

T-test, Wilcoxon rank-sum and Chi-squared tests used to assess between-group differences. IQR = interquartile range, HCW = healthcare worker, BMI = body mass index.

**Fig 1:**
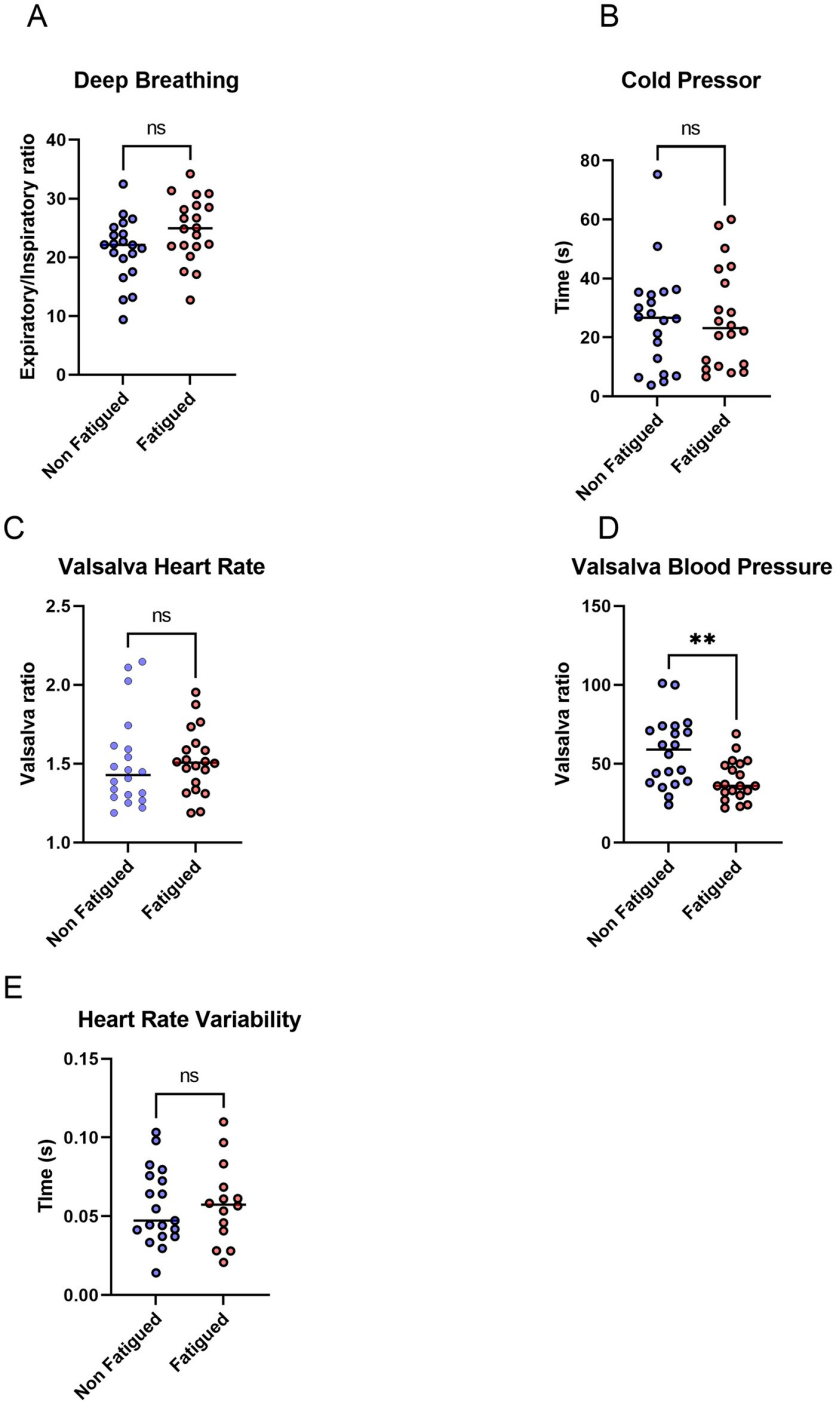
Results of Ewing’s autonomic battery. Results of autonomic testing of fatigued and non-fatigued patients. **(A)** deep breathing **(B)** cold pressor test **(C)** Valsalva heart rate ratio **(D)** Valsalva blood pressure ratio **(E)** heart rate variability. T-test was used to assess differences in deep breathing and heart rate variability. Wilcoxon rank-sum test used to assess differences in cold pressor and Valsalva tests. * p <0.05 ** p <0.01 ***p <0.001 ns = not significant.

All patients underwent autonomic testing as per protocol. There were no differences in the heart rate response to deep breathing (Fig 1A) or time to blood pressure response to cold pressor testing (Fig 1B). The Valsalva manoeuvre demonstrated no difference in the heart rate ratio (Fig 1C) but there was a more marked blood pressure response to Valsalva in the non-fatigued cohort (Fig 1D). There were no differences in heart rate variability prior to active stand (Fig 1E and 1F). The median values and interquartile ranges for these tests were within the normal ranges for both cohorts (S1 Table).

**Fig 2:**
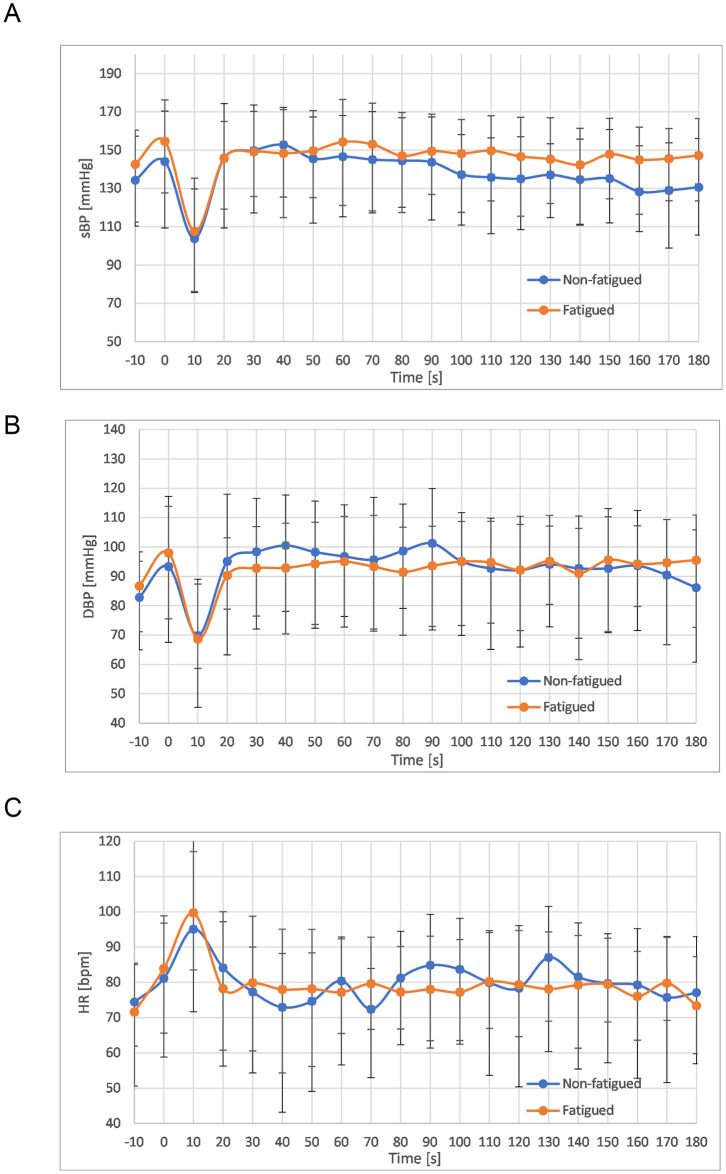
Heart rate and blood pressure changes during active standing. Variation in mean values and standard deviations of **(A)** systolic blood pressure **(B)** diastolic blood pressure **(C)** heart rate from 10 seconds prior to active stand to 180 seconds after active stand. T tests used to assess differences between cohorts at each 10-second timepoint.

**Fig 3:**
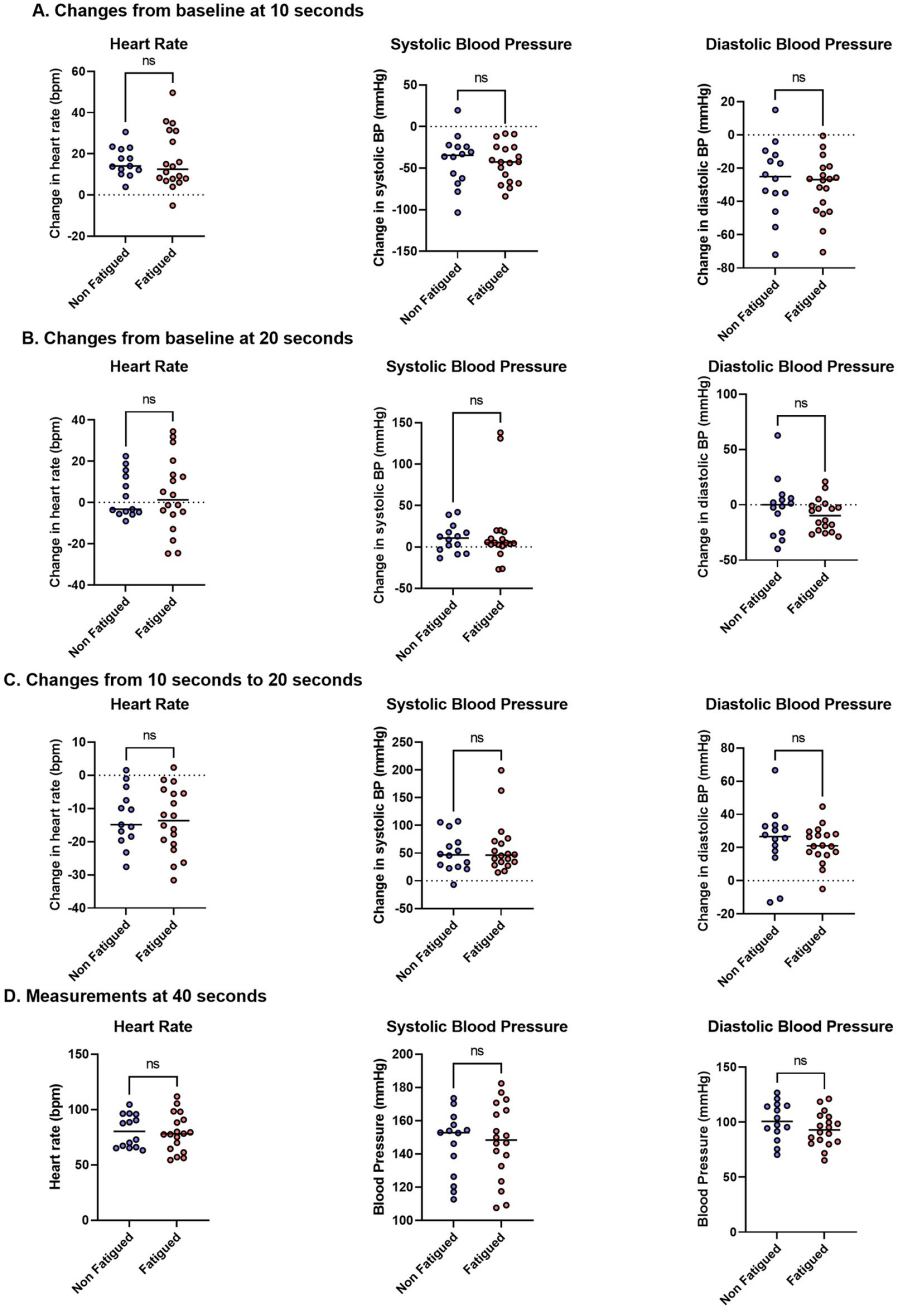
Change in heart rate and blood pressure following an active stand. Changes from baseline measurements in heart rate, systolic blood pressure and diastolic blood pressure shown at **(A)** 10 seconds and **(B)** 20 seconds. Rate of change from 10 seconds to 20 seconds in heart rate and blood pressure are shown in **(C)**. Results at 40 seconds are shown in **(D)**. T-tests and Wilcoxon rank-sum tests used to assess between-group differences. ns = not significant.

Following active standing, 14/20 (70%) of the patients in the fatigued cohort reported at least one of the following symptoms: palpitations, dizziness, feeling lightheaded, or chest discomfort. No patients in the non-fatigued cohort reported any symptoms during active standing. There were no significant differences in HR, systolic BP or diastolic BP between fatigued and non-fatigued individuals at any timepoint during the active stand (Fig 2). Furthermore, there were no differences in the changes in HR, systolic BP or diastolic BP from baseline to 10 seconds (Fig 3A) and 20 seconds (Fig 3B) in both groups, nor were there any differences in change in these values between 10 and 20 seconds (Fig 3C). There was stabilisation of HR and BP at 40 seconds following active standing (Fig 3D). The results of the statistical testing of these measures are shown in S2 Table.

**Fig 4:**
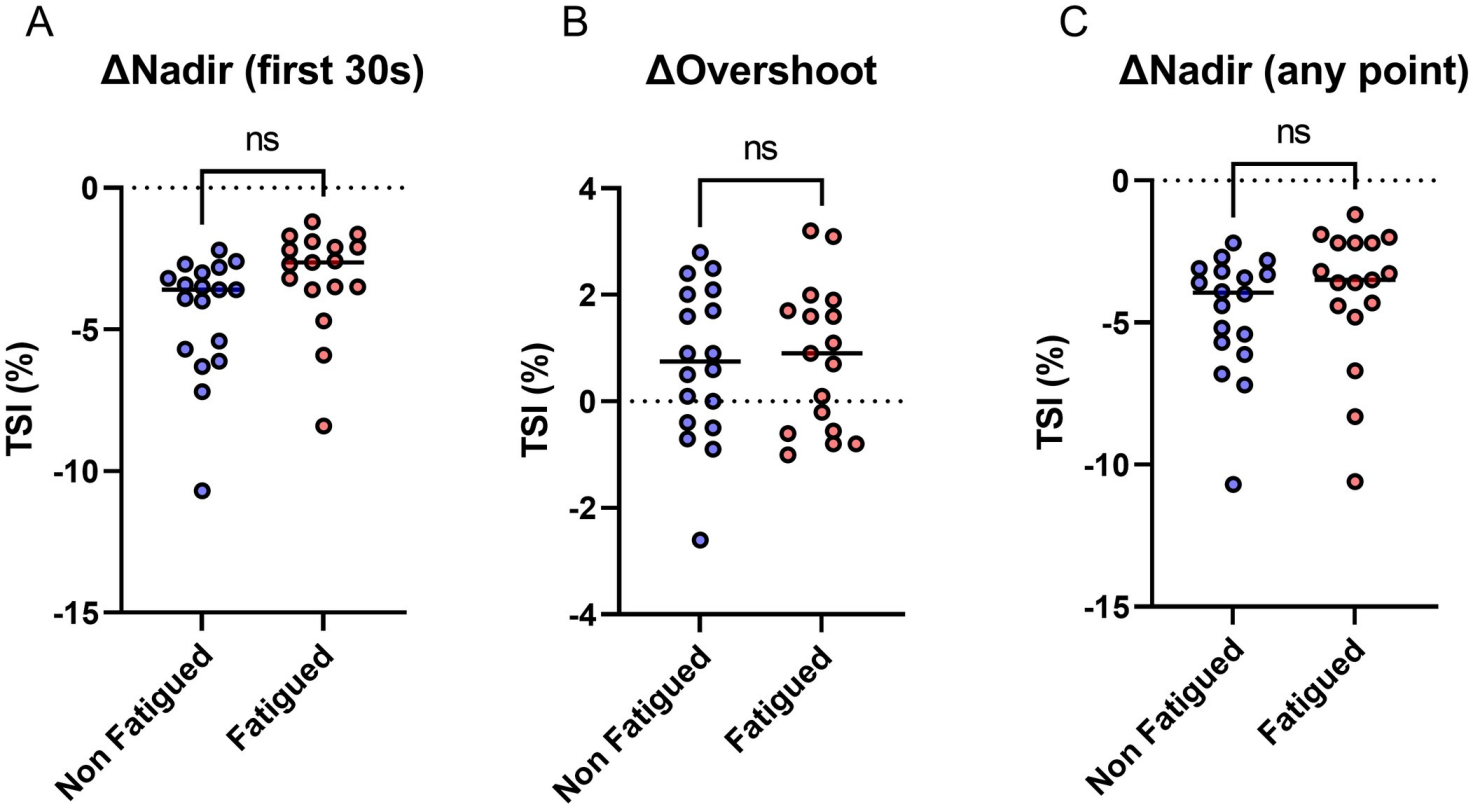
Near-infrared spectroscopy during active standing. Percentage TSI during active standing, showing difference between baseline and **(A)** nadir in first 30 seconds **(B)** maximum overshoot **(C)** nadir at any point. T-test used to assess statistical difference. TSI = tissue saturation index. ns = not significant.

Cerebral blood flow was also assessed during active standing. There were no significant differences between groups in changes from baseline TSI to nadir TSI within 30 seconds of standing, TSI overshoot, or nadir TSI at any point during active standing (Fig 4). The means and standard deviations of these measurements can be found in S2 Table.

All participants completed the GAD7 questionnaire to assess anxiety. Significantly higher levels of anxiety were noted in patients that met the case definition of fatigue versus those that did not (median anxiety score non-fatigued 0 (IQR 0–6) versus fatigued 5.5 (3.5–10.5), *z* -3.14, *p* 0.002).

We next assessed the relationship between total fatigue scores and both autonomic test results and anxiety, as this provides a more granular assessment than a binary fatigue case definition. Following unadjusted and adjusted linear regression, significant associations persisted between total fatigue score and both blood pressure response to Valsalva and anxiety score (Table 2).

**Table 2:** Relationship between fatigue score, autonomic testing and anxiety scores.

Predictor	Model 1		Model 2	
	β coefficient (95% CI)	P value	β coefficient (95% CI)	P value
Deep breathing HR	0.19 (-0.07–0.46)	0.14	0.24 (-0.12–0.59)	0.18
Active stand HR	0.003 (-0.01–0.01)	0.51	0.002 (-0.01–0.01)	0.69
Valsalva HR	0.001 (-0.01–0.01)	0.81	-0.002 (-0.02–0.01)	0.82
Valsalva BP	-1.46 (-2.29 –-0.63)	0.001	-1.89 (-2.98 –-0.81)	0.001
Cold pressor time	-0.42 (-1.23–0.39)	0.30	-0.56 (-1.62–0.50)	0.29
HR variation SD	0.0001 (-0.001–0.001)	0.94	-0.0002 (-0.002–0.001)	0.81
Δ nadir TSI (first 30s)	0.08 (-0.03–0.18)	0.14	0.01 (-0.14–0.16)	0.88
Δ overshoot TSI	0.01 (-0.06–0.08)	0.77	0.01 (-0.11–0.12)	0.91
Δ nadir TSI (any point)	0.03 (-0.09–0.15)	0.59	-0.08 (-0.24–0.08)	0.33
GAD-7 score	0.44 (0.24–0.63)	<0.001	0.54 (0.28–0.78)	<0.001

Linear regression analysis of autonomic tests and anxiety score with Chalder Fatigue Scale– 11 score under Model 1 (unadjusted) and Model 2 (adjusted for age, sex, frailty and need for admission). HR = heart rate; BP = blood pressure; SD = standard deviation; TSI = tissue saturation index; GAD-7 = generalized anxiety disorder-7 questionnaire.

Finally, differences in heart rate variability and blood pressure variability between fatigued and non-fatigued cases were assessed over 24 hours. There were no significant differences between systolic blood pressure, diastolic blood pressure, or heart rate between groups either over the 24-hour monitored period or when divided into day- and night-time measurements. Similarly, there were no differences in MAP between the groups (Table 3).

**Table 3:** 24-hour ambulatory blood pressure monitoring.

	Total cohort (n = 40)	Non-Fatigued (n = 20)	Fatigued (n = 20)	Statistic
**24 hours**				
Systolic BP, mmHg, mean (SD)	110 (10)	109 (10)	110 (11)	*t* -0.33, *p* = 0.75
Systolic BP SD, mmHg, median (IQR)	11.5 (9.8–13.9)	10.1 (9–12.0)	12.6 (10.0–16.4)	*z* -1.76, *p* = 0.08
Diastolic BP, mmHg, mean (SD)	71 (7)	70 (7)	71 (7)	*t* -0.56, *p* = 0.58
Diastolic BP SD, mmHg, median (IQR)	10.5 (3)	9.3 (2.1)	11.4 (3.3)	*t* -1.96, *p* = 0.06
Mean arterial pressure, mmHg, median (IQR)	84 (80–90)	84 (80–87)	83.5 (80–90)	*z* -0.32, *p* = 0.75
Heart rate, bpm, mean (SD)	74.9 (7.8)	75.4 (8.1)	74.6 (7.8)	*t* 0.29, *p* = 0.78
**Day**				
Systolic blood pressure, mmHg, mean (SD)	114 (12)	112 (11)	115 (13)	*t* -0.76, *p* = 0.45
Diastolic blood pressure, mmHg, mean (SD)	74 (8)	73 (7)	75 (8)	*t* -0.97, *p* = 0.34
Mean arterial pressure, mmHg, mean (SD)	89 (8)	87 (7)	90 (9)	*t* -0.94, *p* = 0.36
Pulse pressure, mmHg, median (IQR)	38 (34–45)	39 (34–44)	36.5 (35–47)	*z* -0.14, *p* = 0.89
Heart rate, bpm, mean (SD)	77.5 (8.8)	78.3 (9.5)	76.9 (8.5)	*t* 0.44, *p* = 0.67
**Night**				
Systolic blood pressure, mmHg, mean (SD)	98 (9)	100 (9)	96 (8)	*t* 1.17, *p* = 0.25
Diastolic blood pressure, mmHg, mean (SD)	61 (7)	62 (8)	60 (7)	*t* 0.63, *p* = 0.54
Mean arterial pressure, mmHg, mean (SD)	74 (7)	76 (8)	73 (7)	*t* 1.02, *p* = 0.32
Pulse pressure, mmHg, median (IQR)	37 (5)	38 (4)	36 (5)	*t* 1.22, *p* = 0.23
Heart rate, bpm, mean (SD)	67 (8)	67 (9)	67 (8)	*t* -0.14, *p* = 0.89

T tests and Wilcoxon rank-sum tests used to assess for differences between cohorts. Bonferroni correction applied, with statistical significance considered p <0.01. BP = blood pressure, SD = standard deviation, IQR = interquartile range.

## Discussion

We present comprehensive autonomic assessment of patients with post-COVID fatigue and contextualise the results with matched non-fatigued COVID survivors at a median of 166 days following infection. We find no objective findings of autonomic dysfunction, with no significant pathological differences noted between groups in any of the Ewing’s battery parameters. We report a strong association with post-COVID fatigue and anxiety. This is notable, given that none of the participants had an antecedent diagnosis of anxiety. We also demonstrate a significant symptom burden, with 70% of fatigued patients reporting symptoms at time of the active stand, but these were independent of neurocardiovascular changes. Finally, we demonstrate the impact of post-COVID fatigue on daily function, with 35% of our fatigued cohort not yet returned to full-time employment.

Given that COVID-19 is a novel infection, studies of cardiovascular and autonomic dysfunction following infection are limited. Thus, it is reassuring that we do not seen any evidence of persistent autonomic dysfunction following COVID-19, particularly given that endothelial cells are affected during acute COVID-19 infection. Prior studies in the area of chronic fatigue syndrome/myalgic encephalomyelitis (CFS/ME) have shown a variety of changes in autonomic function. Symptoms of autonomic dysfunction have been shown in subsets of CFS patients [38]. However, objective correlations with symptoms are inconsistently found. CFS has previously been associated with reduced HRV, and it has been suggested that this can be a useful bedside measure for CFS [39]. Orthostatic tachycardia in the absence of hypotension is the hallmark of POTS [40,41]. No patients in our study meet POTS criteria, nor do they demonstrate differences in HRV, suggesting that these changes are not the cause of their ongoing symptoms. Similarly, we see no differences in cerebral blood flow between fatigued and non-fatigued individuals. This is again in contrast to previous studies in CFS and ME, which have been associated with reduced cerebral blood flow [42,43].

The significant difference in blood pressure response to the Valsalva manoeuvre in phase IV is noteworthy. While both non-fatigued and fatigued patients have responses that are within normal physiological limits, the response is more marked in those without fatigue. Discrepancies in expiratory effort can lead to this occurring [44]. Volume status may also affect the Valsalva response [45]. Other known confounders such as age and sex were controlled for during analysis. It is unclear whether the difference seen is of biological significance, given that the results are within normal limits.

The discrepancies in our study with previous findings in the area of CFS and ME suggest that post-COVID fatigue is distinct from these conditions, despite sharing similar clinical characteristics. Similarly, we did not see changes that would fit with a diagnosis of POTS. The robust physiological assessments performed in our study clearly demonstrate the absence of significant dysautonomia in post-COVID fatigue. Furthermore, our population is representative of those reported to be most at-risk from developing *long COVID*, namely young females [19,46,47]. The high proportion of females in our study replicate the clinical population seen, and supports the generalisation of these results to the larger post-COVID population.

We do however see a strong association between post-COVID-19 fatigue and anxiety. This is of particular note, given that none of the patients in this study had a pre-existing diagnosis of anxiety, nor were they taking any anxiolytic medications. The association between chronic fatigue and anxiety is well-described [48,49]. The aetiology of anxiety in the setting of chronic fatigue appears to be multi-factorial, with both genetic and autonomic causes proposed [50,51]. Socioeconomic factors such as loss of income due to discontinuation of employment have also been linked with the development of anxiety in fatigue syndromes [52]. This is noteworthy, given that 35% of our fatigued cohort have not returned to full-time employment. This finding suggests that patients with post-COVID fatigue should be investigated for concurrent anxiety and managed accordingly.

Our study has some limitations of note. Our sample cohort is not large enough to investigate associations with each individual symptom reported, and these are grouped together. However, our cohorts are well-matched, and this is evident by the limited effect multiple linear regression has on the associations investigated. We did not measure serological markers of the adrenergic system. Ewing’s battery is however a robust non-invasive measure of this system. The GAD7 is a rapid screening tool, and more detailed anxiety assessments are warranted in future studies. Furthermore, our cohort has a large proportion of healthcare workers, and anxiety may be due to occupational stress during the pandemic. Finally, we did not investigate alternative causes of symptoms that mimic autonomic dysfunction, such as vestibular dysfunction. We would suggest that this is an area worthy of further study.

Our study demonstrates the significant burden of fatigue at a median interval of 166 days following COVID-19 infection, with significant associated anxiety as well as failure to return to employment. We demonstrate symptoms of autonomic dysfunction at active standing without a physiological cardiovascular cause. These findings suggest that autonomic dysfunction is not a significant contributor to post-COVID-19 fatigue.

## Supporting information

S1 Table Deep breathing, Valsalva and cold pressor results between cohorts. T-test and Wilcoxon rank-sum tests used to assess between-group differences. ms = milliseconds; SD = standard deviation. IQR = interquartile range.
(DOCX)

S2 Table Comparison of active stand cardiovascular parameters between groups. Changes in heart rate, blood pressure and cerebral oxygenation during active stand, as well as heart rate variability prior to active stand. T-test and Wilcoxon rank-sum used to assess between-group differences. HR = heart rate, SBP = systolic blood pressure, DBP = diastolic blood pressure. Δ = change from baseline measurement. Δ1020 = change from 10 seconds to 20 seconds. IQR = interquartile range. SD = standard deviation. TSI = tissue saturation index.
(DOCX)
