# Supplementary material for: Fatigue following COVID-19 infection is not associated with autonomic dysfunction
Source: PLoS One. 2021 Feb 25;16(2):e0247280. doi: 10.1371/journal.pone.0247280 (PMC7906457; doi:10.1371/journal.pone.0247280)
Supplement: S1 Table — T-test and Wilcoxon rank-sum tests used to assess between-group differences. ms = milliseconds; SD = standard deviation. IQR = interquartile range. (DOCX) [file pone.0247280.s001.docx]

**Supplemental Table 1:** Deep breathing, Valsalva and cold pressor results between cohorts

|  | Cohort (n=40) | Non-Fatigued (n=20) | Fatigued (n=20) | Statistic |
| --- | --- | --- | --- | --- |
| Deep Breathing HR response mean (SD) | 23.10 (5.6) | 21.34 (5.43) | 24.77 (5.44) | *t* -1.99, *p* 0.053 |
| Valsalva R-R median (IQR) | 1.48 (1.31 – 1.60) | 1.43 (1.30 – 1.60) | 1.51 (1.36 – 1.61) | *z* -0.76, *p* 0.45 |
| Valsalva BP response median (IQR) | 44.5 (34 – 62) | 59 (38.5 – 72.5) | 36 (31 – 49.5) | *z* 2.84, *p* 0.005 |
| Cold pressor test time median (IQR) | 25.66 (10.61 – 35.46) | 26.68 (10.19 – 34.95) | 23.18 (10.61 – 40.83) | *z* -0.14, *p* 0.89 |
| Heart rate variability SD, ms, mean (SD) | 0.057 (0.024) | 0.056 (0.024) | 0.058 (0.026) | *t* -0.22, *p* 0.83 |

T-test and Wilcoxon rank-sum tests used to assess between-group differences. ms=milliseconds; SD=standard deviation. IQR=interquartile range.
