# Supplementary material for: Fatigue following COVID-19 infection is not associated with autonomic dysfunction
Source: PLoS One. 2021 Feb 25;16(2):e0247280. doi: 10.1371/journal.pone.0247280 (PMC7906457; doi:10.1371/journal.pone.0247280)
Supplement: S2 Table — Changes in heart rate, blood pressure and cerebral oxygenation during active stand, as well as heart rate variability prior to active stand. T-test and Wilcoxon rank-sum used to assess between-group differences. HR = heart rate, SBP = systolic blood pressure, DBP = diastolic blood pressure. Δ = change from baseline measurement. Δ1020 = change from 10 seconds to 20 seconds. IQR = interquartile range. SD = standard deviation. TSI = tissue saturation index. (DOCX) [file pone.0247280.s002.docx]

**Supplemental Table 2:** Comparison of active stand cardiovascular parameters between groups

|  | Total cohort (n=40) | Non-Fatigued (n=20) | Fatigued (n=20) | Statistic |
| --- | --- | --- | --- | --- |
| Active stand HR response median (IQR) | 1.17 (1.09 – 1.25) | 1.15 (1.05 – 1.2) | 1.20 (1.12 – 1.30) | *z* -1.43, *p* 0.15 |
| ΔHR10, bpm, median (IQR) | 13.80 (8.16 – 23.20) | 13.91 (10.13 – 22.24) | 12.48 (7.82 – 31.13) | *z* 0.04, *p* 0.97 |
| ΔSBP10, mmHg, mean (SD) | -42 (26) | -41 (31) | -43 (23) | *t* 0.30, *p* 0.77 |
| ΔDBP10, mmHg, mean (SD) | -29 (20) | -26 (22) | -31 (18) | *t* 0.66, *p* 0.52 |
| ΔHR20, bpm, mean (SD) | 2.2 (16.7) | 0.4 (15.2) | 3.5 (18.0) | *t* -0.52, *p* 0.61 |
| ΔSBP20, mmHg, mean (SD) | 15 (35) | 11 (17) | 18 (44) | *t* -0.56, *p* 0.58 |
| ΔDBP20, mmHg, mean (SD) | -6 (20) | -2 (26) | -9 (15) | *t* -0.56, *p* 0.58 |
| ΔHR1020, bpm, median (IQR) | -13.50 (-19.53 – -4.86) | -12.57 (-18.45 – -3.44) | -13.63 (-20.75 – -5.45) | *z* 0.53, *p* 0.59 |
| ΔSBP1020, mmHg, median (IQR) | 46.43 (28.63 – 70.56) | 46.91 (25.26 – 69.40) | 46.33 (34.24 – 71.72) | *z* -0.30, *p* 0.76 |
| ΔDBP1020, mmHg, median (IQR) | 25.39 (16.67 – 30.66) | 26.63 (17.97 – 32.86) | 21.02 (15.94 – 28.09) | *z* 0.04, *p* 0.97 |
| SBP at 40 seconds, mmHg, mean (SD) | 6 (19) | 9 (16) | 3 (22) | *t* 0.82, *p* 0.42 |
| DBP at 40 seconds, mmHg, mean (SD) | 11 (15) | 15 (11) | 7 (17) | *t* 1.41, *p* 0.17 |
| HR at 40 seconds, median (IQR) | 6.23 (-4.01 – 14.75) | 6.23 (-0.13 – 14.98) | 6.46 (-5.2 – 13.53) | *z* 0.76, *p* 0.48 |
| Δ nadir TSI (first 30s), %, mean (SD) | -3.81 (2.05) | -4.44 (2.13) | -3.15 (1.80) | *t* -1.97, *p* 0.06 |
| Δ overshoot TSI , %, mean (SD) | 0.77 (1.38) | 0.72 (1.44) | 0.82 (1.36) | *t* -0.21, *p* 0.58 |
| Δ nadir TSI (any point), %, mean (SD) | -4.33 (2.28) | -4.65 (2.10) | -4.00 (2.47) | *t* -0.84, *p* 0.40 |

Changes in heart rate, blood pressure and cerebral oxygenation during active stand, as well as heart rate variability prior to active stand. T-test and Wilcoxon rank-sum used to assess between-group differences. HR=heart rate, SBP=systolic blood pressure, DBP=diastolic blood pressure. Δ=change from baseline measurement. Δ1020=change from 10 seconds to 20 seconds. IQR=interquartile range. SD=standard deviation. TSI=tissue saturation index
